# All Three AKT Isoforms Can Upregulate Oxygen Metabolism and Lactate Production in Human Hepatocellular Carcinoma Cell Lines

**DOI:** 10.3390/ijms25042168

**Published:** 2024-02-11

**Authors:** Ling-Yu Tian, Daniel J. Smit, Nadezhda V. Popova, Stefan Horn, Lis Noelia Velasquez, Samuel Huber, Manfred Jücker

**Affiliations:** 1 Institute of Biochemistry and Signal Transduction, University Medical Center Hamburg-Eppendorf, Martinistraße 52, 20246 Hamburg, Germany; l.tian.ext@uke.de (L.-Y.T.); d.smit@uke.de (D.J.S.); npopova@gmail.com (N.V.P.); 2 Beijing Key Surgical Basic Research Laboratory of Liver Cirrhosis and Liver Cancer, Department of Hepatobiliary Surgery, Peking University People’s Hospital, Beijing 100044, China; 3 Research Department Cell and Gene Therapy, Department of Stem Cell Transplantation, University Medical Center Hamburg-Eppendorf, 20246 Hamburg, Germany; s.horn@uke.de; 4 I. Department of Medicine, University Medical Center Hamburg-Eppendorf, 20246 Hamburg, Germany; l.velasquez@uke.de (L.N.V.); s.huber@uke.de (S.H.); 5 Hamburg Center for Translational Immunology, University Medical Center Hamburg-Eppendorf, 20246 Hamburg, Germany

**Keywords:** AKT signaling, AKT isoforms, oxygen metabolism, lactate metabolism, extracellular acidification, glycolysis, Warburg effect, HCC metabolism, HCC treatment

## Abstract

Hepatocellular carcinoma (HCC), the main pathological type of liver cancer, is related to risk factors such as viral hepatitis, alcohol intake, and non-alcoholic fatty liver disease (NAFLD). The constitutive activation of the PI3K/AKT signaling pathway is common in HCC and has essential involvement in tumor progression. The serine/threonine kinase AKT has several downstream substrates, which have been implicated in the regulation of cellular metabolism. However, the contribution of each of the three AKT isoforms, i.e., AKT1, AKT2 and AKT3, to HCC metabolism has not been comprehensively investigated. In this study, we analyzed the functional role of AKT1, AKT2 and AKT3 in HCC metabolism. The overexpression of activated AKT1, AKT2 and AKT3 isoforms in the human HCC cell lines Hep3B and Huh7 resulted in higher oxygen consumption rate (OCR), ATP production, maximal respiration and spare respiratory capacity in comparison to vector-transduced cells. Vice versa, lentiviral vector-mediated knockdowns of each AKT isoform reduced OCR in both cell lines. Reduced OCR rates observed in the three AKT isoform knockdowns were associated with reduced extracellular acidification rates (ECAR) and reduced lactate production in both analyzed cell lines. Mechanistically, the downregulation of OCR by AKT isoform knockdowns correlated with an increased phosphorylation of the pyruvate dehydrogenase on Ser232, which negatively regulates the activity of this crucial gatekeeper of mitochondrial respiration. In summary, our data indicate that each of the three AKT isoforms is able to upregulate OCR, ECAR and lactate production independently of each other in human HCC cells through the regulation of the pyruvate dehydrogenase.

## 1. Introduction

Hepatocellular carcinoma (HCC) is the second leading cause of cancer death in East Asia and the sixth leading cause of cancer death in Western countries. The similarity between incidence (906,000 per year) and mortality (830,000 deaths per year) underlines the dismal prognosis associated with this disease [1,2]. The leading risk factors of HCC are hepatitis, virus infection, auto-immune diseases, drug- and non-drug-related toxicity, as well as non-alcohol fatty liver disease (NAFLD) [3]. Increasing evidence suggests the strong association between metabolic factors and HCC prevalence [3]. Metabolic reprogramming is one of the most substantial tumor hallmarks [4]. Recently, tremendous attention has been brought to the implication of deregulated metabolism on HCC carcinogenesis [5].

Frequently, the alteration of intracellular signaling pathways has been observed in HCC and has been associated with tumor progression. The phosphoinositide 3-kinase (PI3K), AKT and mammalian target of rapamycin (mTOR) pathway are among these oncogenic signal transduction pathways in HCC [6]. The PI3K/AKT/mTOR signaling pathway regulates crucial cellular processes, including cell survival, metastasis and metabolism. It can be aberrantly activated through various mechanisms, including genomic alterations and mutations of PIK3CA, AKT and mTOR or a loss of the tumor suppressor phosphatase and tension homolog (PTEN) [7]. Cancer with activated PI3K/AKT signaling has been demonstrated to present as an aggressive phenotype, and the activation of PI3K/AKT signaling has been indicated as a significant risk factor for early recurrence and poor prognosis in liver cancer patients [8,9,10]. The PI3K/AKT signaling networks have multifarious downstream influences on cellular metabolism, via both the direct regulation of nutrient transporters and metabolic enzymes as well as the regulation of transcription factors that control the expression of key elements of metabolic pathways [11]. Some downstream molecules of the PI3K/AKT/mTOR signaling pathway are particularly increased in HCC patients with elevated 2[18F]fluoro-2-deoxy-d-glucose (FDG) uptake, implying that the activation of this pathway might regulate the HCC metabolism [12].

The AKT family has three members (AKT1/PKBα, AKT2/PKBβ and AKT3/PKBγ) sharing a high structural homology, but studies on AKT isoform-specific knockout mice suggest that AKT signaling diversity might in part be due to different functions of the three AKT family members [13]. The AKT1 knockout (KO) mice are growth-restricted but have no metabolic irregularities and AKT2 KO mice are glucose intolerant and show systemic insulin resistance, whereas AKT3 KO mice have decreased brain size but regular glucose homeostasis [14]. Recently, several studies showed functional roles of the three AKT isoforms in multiple solid tumors, demonstrating that different isoforms have distinct functions in tumors [15,16,17]. In tumor metabolic studies, AKT1 activation was associated with the accumulation of aerobic glycolysis metabolites in prostate cancer [18]. Moreover, the AKT2 phosphorylation of hexokinase 2 has been demonstrated to enhance hexokinase activity and lactic acid production in colon cancer [19]. Furthermore, for AKT3 knockdown, it has been demonstrated to cause mitochondrial dysfunction in human lung cancer cells [20].

The liver plays a crucial role in the body’s metabolic homeostasis and is significantly involved in the regulation of various metabolic processes. Nevertheless, the functional role of the three AKT isoforms in metabolic regulation has never been simultaneously investigated in HCC [21]. Therefore, we aimed to clarify the metabolic roles of three AKT isoforms, AKT1, AKT2 and AKT3, in human HCC cell lines and to provide a rationale for AKT inhibition in HCC patients. Our data demonstrate that all three AKT family members can upregulate oxygen metabolism and extracellular acidification in human HCC cell lines.

## 2. Results

### 2.1. Generation of Stable Knockdowns and Ectopic Expression of Activated AKT1, AKT2 and AKT3 Mutants in Human HCC Cell Lines

**Figure 1:**
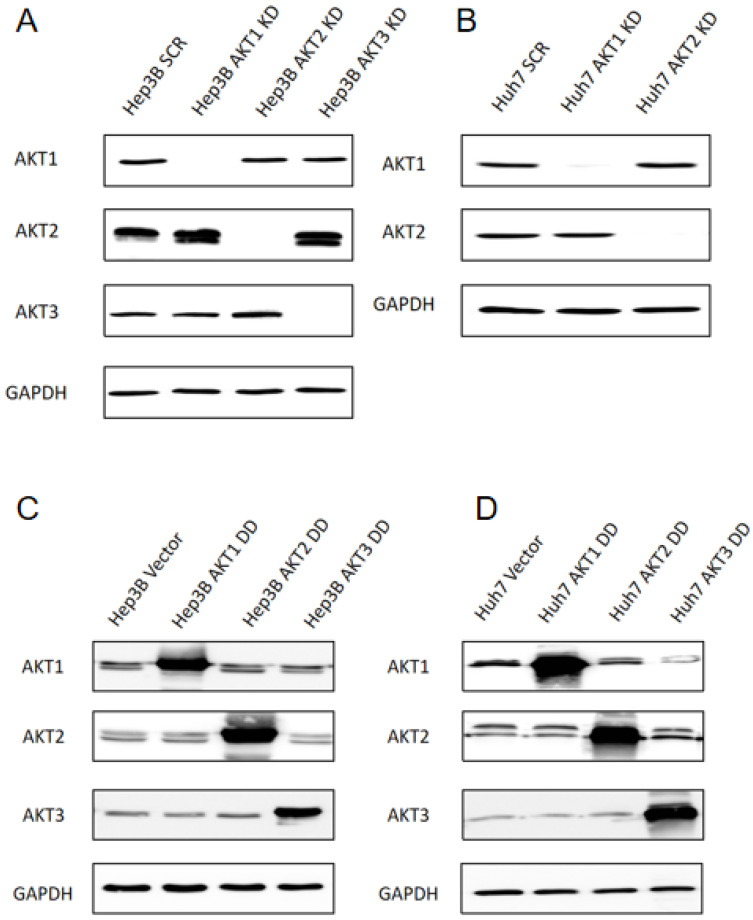
Stable knockdown or overexpression of AKT isoforms in HCC cell lines. Knockdown of AKT isoforms was conducted by lentiviral transduction with AKT-isoform-specific shRNAs in Hep3B (**A**) or Huh7 (**B**) cells. Overexpression of AKT1, AKT2 and AKT3 isoforms upon transduction with constitutively activated AKT lentiviral vectors in Hep3B (**C**) or Huh7 (**D**) cells. Protein expression levels of AKT isoforms were examined by Western blot analysis using AKT-isoform-specific antibodies. The experiments were repeated three times and the representative results are shown.

Our previous findings have shown that Hep3B and Huh7 cells express AKT1 and AKT2, but AKT3 expression is restricted to Hep3B cells [15]. Therefore, stable knockdowns of AKT1, AKT2 and AKT3 were introduced in Hep3B cells (Figure 1A) as well as AKT1 and AKT2 knockdowns in Huh7 cells (Figure 1B). The knockdown efficiency in Hep3B is 98.9%, 95.3% and 98.9% for AKT1, AKT2 and AKT3, respectively. For Huh7, the knockdown efficiency of AKT1 and AKT2 is 91.4% and 98.2%, respectively (Supplementary Figure S1). In addition, constitutively activated AKT isoforms were strongly overexpressed in Hep3B and Huh7 cells by the lentiviral transduction of vectors with constitutively activated AKT by amino acid exchange to aspartic acid (i.e., AKT1 T308D/S473D, AKT2 T309D/S474D, AKT3 T305D/S472D) (Figure 1C,D). AKT1 DD, AKT2 DD and AKT3 DD displayed 4.4, 8.2 and 21.8-fold higher expression in comparison to the Hep3B parental cell line, respectively. In Huh7 cells, AKT1 DD, AKT2 DD and AKT3 DD displayed 15.5, 7.5 and 101.4-fold higher expression than the parental HuH7 cell line, respectively (Supplementary Figure S2).

### 2.2. The Knockdown of AKT1, AKT2 and AKT3 Inhibits the Oxygen Consumption Rate of HCC Cells

**Figure 2:**
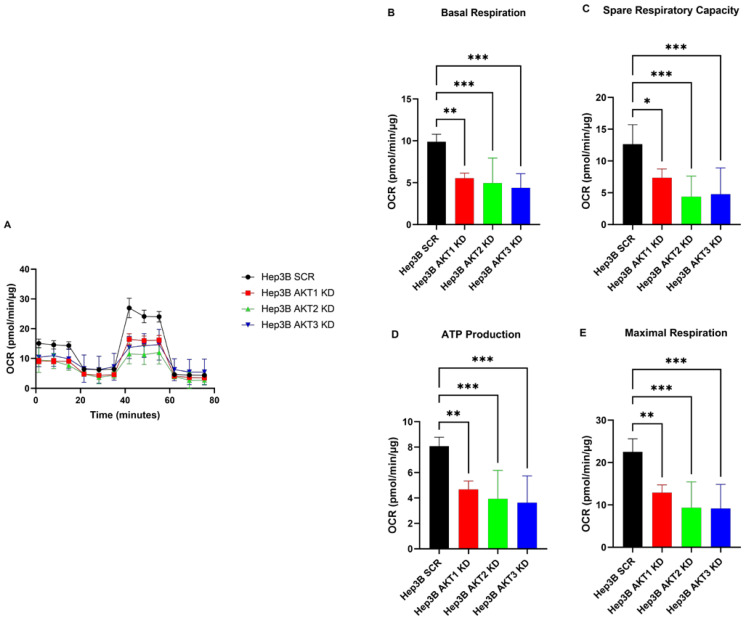
Effect of AKT1, AKT2 and AKT3 silencing on oxygen metabolism in the Hep3B cell line. (**A**) Knockdown of AKT1, AKT2 and AKT3 reduced the oxygen consumption rate (OCR) in HCC cell lines Hep3B. (**B**) Summary statistics of Basal rate of respiration. (**C**) Summary statistics of spare respiratory capacity. (**D**) Summary statistics of oligomycin-sensitive respiration (ATP production). (**E**) Summary statistics of uncoupled respiration (maximal respiratory capacity). The experiments were repeated six times and the pooled results are shown. *p*-values were calculated with ordinary one-way ANOVA with Dunnett’s multiple comparison test. * *p* < 0.05, ** *p* < 0.01, *** *p* < 0.001.

**Figure 3:**
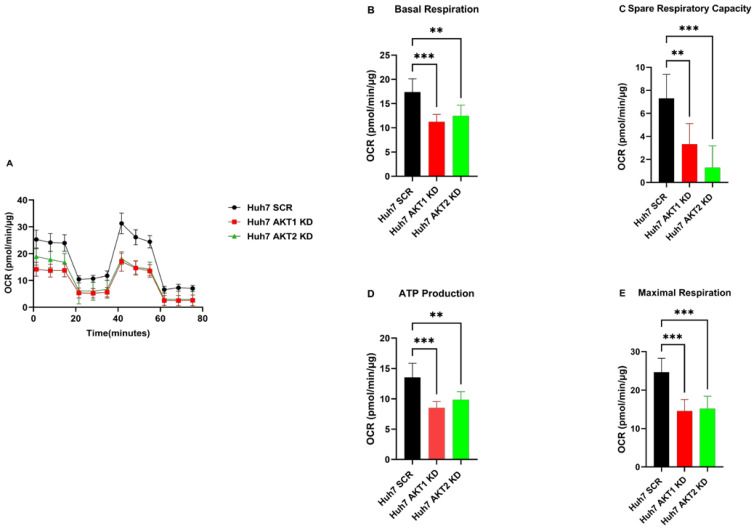
Effect of AKT1 and AKT2 silencing on the oxygen metabolism in Huh7 cell lines. (**A**) Knockdown of AKT1 and AKT2 reduced the oxygen consumption rate (OCR) in the HCC cell line Huh7. (**B**) Summary statistics of Basal rate of respiration. (**C**) Summary statistics of spare respiratory capacity. (**D**) Summary statistics of oligomycin sensitive respiration (ATP production). (**E**) Summary statistics of uncoupled respiration (maximal respiratory capacity). The experiments were repeated six times and the pooled results are shown. *p*-values were calculated with ordinary one-way ANOVA with Dunnett’s multiple comparison test. ** *p* < 0.01, *** *p* < 0.001.

We first determined the proliferative behavior of HCC cells with AKT isoform knockdowns using the IncuCyte Zoom live cell imaging system. The knockdown of AKT1 and knockdown of AKT2 decreased the proliferation of both HCC cells, i.e., Hep3B and HuH7, whereas the knockdown of AKT3 did not show a significant alteration in proliferation in comparison to the SCR control in Hep3B cells (Supplementary Figure S3). We determined the real-time state of the oxygen metabolism profiles of AKT1, AKT2 and AKT3 knockdown of HCC cell lines using the Seahorse XF Cell Mito Stress Test. We observed that Hep3B cell lines with AKT1, AKT2 and AKT3 knockdown exhibited lower oxygen consumption rates (OCR) than Hep3B cells after transduction with the scrambled control vector (Figure 2A). A detailed data analysis further showed that the AKT1, AKT2 and AKT3 knockdown cells had a significantly lower basal OCR, maximal respiration and ATP production compared to the control cells. Moreover, AKT1, AKT2 and AKT3 knockdown decreased the spare respiratory capacity, which means the percentage of total oxygen consumptive capacity that is not utilized and can be reserved in reacting to raised metabolic demands (Figure 2B–E). Similarly, significantly lower basal OCR, maximal respiration and ATP production as well as spare respiratory capacity were found in Huh7 cells after the knockdown of AKT1 or AKT2 in comparison to the control Huh7 cells (Figure 3). In conclusion, the OCR parameters (basal respiration, maximal respiration, spare respiratory capacity and ATP production) were significantly reduced after each individual AKT isoform knockdown in both HCC cell lines.

### 2.3. AKT1, AKT2 and AKT3 Promote OCR in Hep3B and Huh7

**Figure 4:**
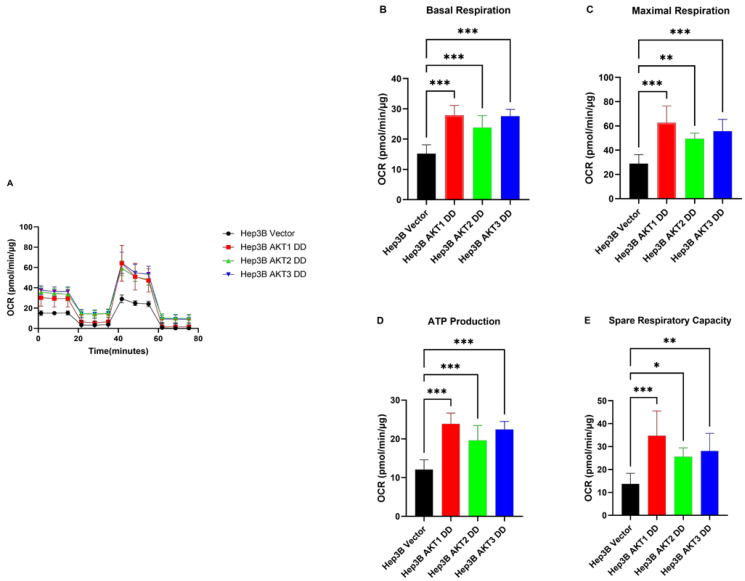
Effect of AKT1, AKT2 and AKT3 activation on the oxygen metabolism of the Hep3B cell lines. (**A**) Overexpression of constantly activated AKT1, AKT2 and AKT3 increased the oxygen consumption rate (OCR) in HCC cell lines Hep3B. (**B**) Summary statistics of Basal rate of respiration in Hep3B. (**C**) Summary statistics of spare respiratory capacity in Hep3B. (**D**) Summary statistics of oligomycin-sensitive respiration (ATP production) in Hep3B. (**E**) Summary statistics of uncoupled respiration (maximal respiratory capacity) in Hep3B. The experiments were repeated six times in Hep3B cells and the pooled results are shown. *p*-values were calculated with ordinary one-way ANOVA with Dunnett’s multiple comparison test. * *p* < 0.05, ** *p* < 0.01, *** *p* < 0.001.

**Figure 5:**
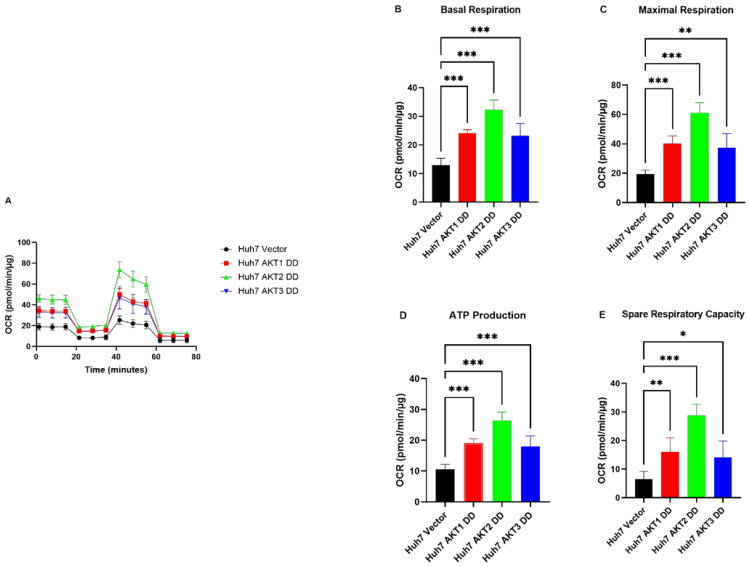
Effect of AKT1, AKT2 and AKT3 activation on the oxygen metabolism of the Huh7 cell lines. (**A**) Overexpression of constantly activated AKT1, AKT2 and AKT3 increased the oxygen consumption rate (OCR) in HCC cell lines Huh7. (**B**) Summary statistics of Basal rate of respiration in Huh7. (**C**) Summary statistics of spare respiratory capacity in Huh7. (**D**) Summary statistics of oligomycin sensitive respiration (ATP production) in Huh7. (**E**) Summary statistics of uncoupled respiration (maximal respiratory capacity) in Huh7. The experiments were repeated five times in Huh7 cells and the pooled results are shown. *p*-values were calculated with ordinary one-way ANOVA with Dunnett’s multiple comparison test. * *p*< 0.05, ** *p* < 0.01, *** *p* < 0.001.

Next, we analyzed whether the observed inhibitory effect on OCR by AKT isoform knockdowns can be reversed by the expression of activated AKT isoforms. We observed that Hep3B cells with activated AKT1, AKT2 and AKT3 variants exhibited higher OCR than control cells (Figure 4A). Moreover, basal OCR, maximal respiration, ATP production and spare respiratory capacity (Figure 4B–E) were significantly higher in mutant AKT1-, AKT2- and AKT3-overexpressing cells than in corresponding control cells. Likewise, we found significantly higher basal OCR, maximal respiration and ATP production, as well as spare respiratory capacity, in the constitutively activated AKT1-, AKT2- and AKT3-overexpressing Huh7 cells compared to the corresponding vector control cells (Figure 5). These data indicate that the activation of AKT1, AKT2 and AKT3 can stimulate all mitochondrial parameters, which contributes to the activation of mitochondrial oxidative phosphorylation in HCC cells.

### 2.4. AKT Isoforms Regulate the Phosphorylation of Pyruvate Dehydrogenase

**Figure 6:**
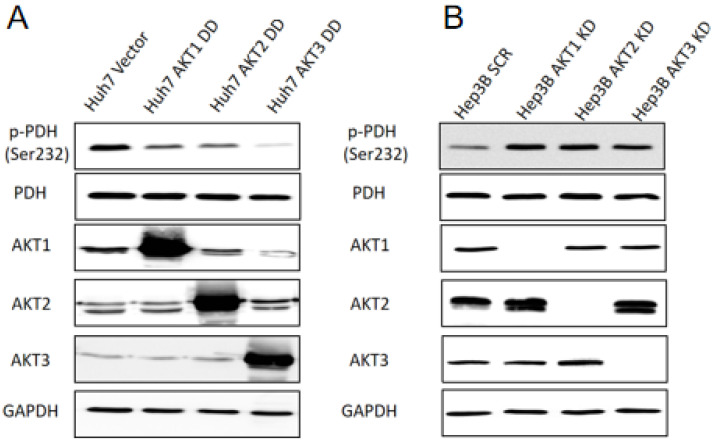
The impact of single AKT isoforms on the inhibitory phosphorylation of PDH at Ser232 in HCC cells. Expression of AKT isoforms, levels of pyruvate dehydrogenase (PDH) phosphorylated at the inhibitory residue Ser232 (p-PDH) as well as total PDH expression were analyzed by Western blot (**A**) after the expression of constitutively activated AKT isoforms in Huh7 cells and (**B**) following the knockdown of individual AKT isoforms in Hep3B cells. The experiments were repeated three times and the representative results are shown.

To gain mechanistic insight into the regulation of AKT-mediated oxygen consumption in HCC cells, we examined the phosphorylation of the pyruvate dehydrogenase (PDH) complex, which is a major regulator of the mitochondrial tricarboxylic acid cycle (TCA) that produces the precursors for the oxidative phosphorylation, i.e., NADH and FADH_2_. As shown in Figure 6, the overexpression of activated AKT1, AKT2 and AKT3 mutants in Huh7 cells resulted in decreased inhibitory phosphorylation at Ser232 (Figure 6A). Conversely, the knockdown of AKT1, AKT2 and AKT3 in Hep3B cells resulted in an increase in Ser232 phosphorylation of PDH (Figure 6B).

### 2.5. The Effects of Three AKT Isoforms on ECAR

**Figure 7:**
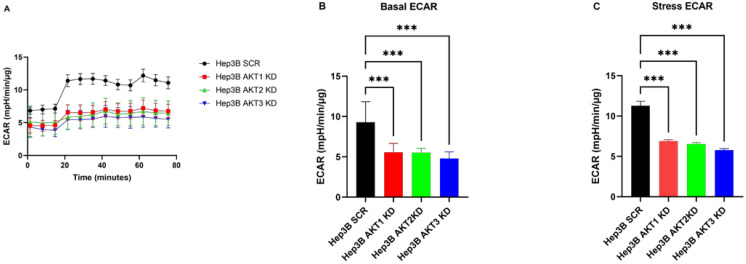

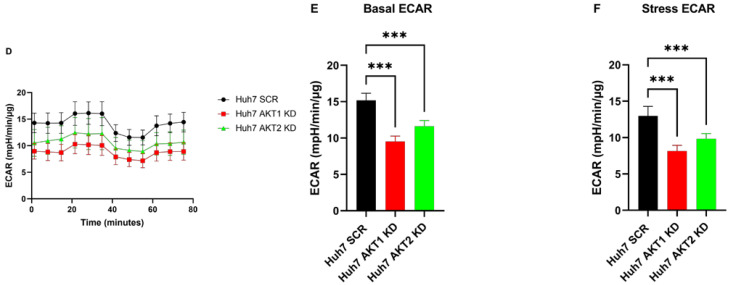
Knockdown of AKT1, AKT2 and AKT3 suppresses ECAR in HCC cell lines. (**A**,**D**) Extracellular acidification rate (ECAR) measured by extracellular flux analysis. (**B**,**E**) Summary statistics of basal ECAR. (**C**,**F**) Summary statistics of stress ECAR. The experiments were repeated six times and the pooled results are shown. *p*-values were calculated with ordinary one-way ANOVA with Dunnett’s multiple comparison test *** *p* < 0.001.

To further clarify the detailed differences in cell metabolism caused by the AKT isoforms, extracellular acidification rates (ECARs) were assessed by a cell flux analyzer. To generate a stressed metabolic phenotype that demonstrates the maximal metabolic potential of the cells, ECARs were estimated before (basal) and after (stressed) the injection of the ATP synthetase inhibitor oligomycin and the mitochondrial de-coupler FCCP. In Hep3B cells, basal and stressed ECAR were significantly lower in the AKT1, AKT2 and AKT3 knockdown as compared to the corresponding control cells (Figure 7A–C). In line with our findings in Hep3B, the knockdown of AKT1 and AKT2 decreased both basal and stressed ECAR in Huh7 cell lines (Figure 7D–F). Taken together, we demonstrated that the knockdown of AKT isoforms decreased both ECAR and OCR in HCC cell lines.

### 2.6. The Knockdown of AKT Isoforms Is Associated with Decreased Extracellular Lactate Production

**Figure 8:**
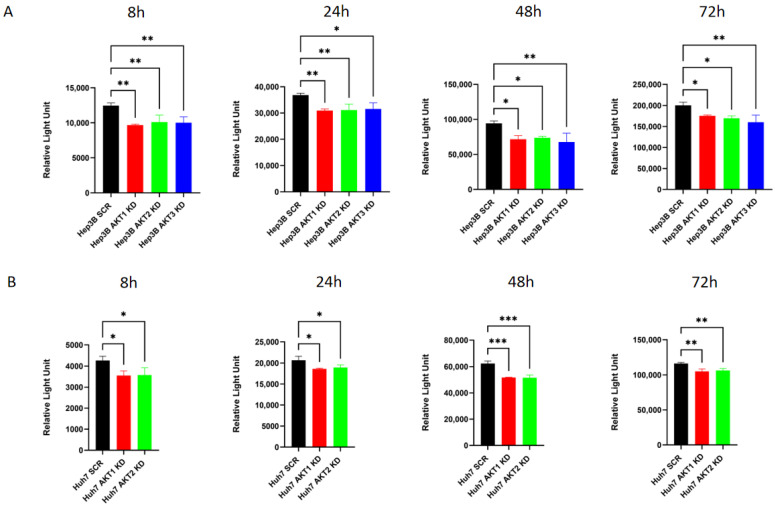
The effects of AKT1, AKT2 and AKT3 on lactic acid production in HCC cells were evaluated using the lactate assay. (**A**) Stable knockdowns of AKT1, AKT2 and AKT3 were generated in (**A**) Hep3B and (**B**) Huh7 cells. Thereafter, the cells were cultured in lactate-free medium for the indicated times and lactate levels were determined. The experiments were repeated three times and the pooled results are shown. *p*-values were calculated with ordinary one-way ANOVA with Dunnett’s multiple comparison test. * *p* < 0.05, ** *p* < 0.01, *** *p* < 0.001.

As lactate contributes to the ECAR, we analyzed the possibility that the observed AKT-mediated effect on the ECAR is due to an AKT-dependent regulation of lactate. Therefore, lactate secretion was measured in the culture medium of Hep3B and Huh7 cells with AKT isoform knockdowns after culturing the cells for 8, 24, 48 and 72 h in fresh medium without lactate. A significant reduction in lactate secretion was found in Hep3B cells after AKT1, AKT2 and AKT3 knockdown (Figure 8A). Consistently, a significant decrease in the extracellular lactate was observed after the knockdown of AKT1 and AKT2 in Huh7 cells (Figure 8B).

## 3. Discussion

In this work, we provide evidence that each of the three AKT isoforms is involved in the regulation of oxygen consumption, extracellular acidification and lactate production in human HCC cells. The overexpression of activated AKT1, AKT2 and AKT3 isoforms resulted in a higher oxygen consumption rate (OCR), ATP production, maximal respiration and spare respiratory capacity in both HCC cell lines examined. Vice versa, lentiviral vector-mediated knockdowns of each AKT isoform reduced OCR, which was associated with reduced extracellular acidification rates (ECAR) and reduced lactate production. Consistent with our findings, lactate production was inhibited by blocking AKT1/GSK3β (glycogen synthase kinase 3 beta) signaling in ovarian cancer cells after treatment with the VEGFR2 kinase inhibitor apatinib [22]. In colon cancer, it has been demonstrated that AKT2 can interact with and phosphorylate hexokinase 2 (HK2), the rate-limiting enzyme in glycolysis. The phosphorylation of HK2 at T473 was observed to raise hexokinase activity and lactic acid production [19]. Osteosarcoma cell glycolysis, proliferation, migration and invasion were suppressed by decreasing glycolysis-related proteins and migration-related proteins via the inhibition of c-MET and AKT3/mTOR [23]. In addition, the knockdown of AKT1 and AKT2 reduced lactate production in the prostatic adenocarcinoma cell line PC3 [24]. Furthermore, the targeted inhibition of AKT1 and AKT3 prevented glycolysis-related enzyme activation, significantly blocked the production of lactate and diminished the migration and invasion of chemoresistant colon cancer cells [25]. All these data are consistent with our findings in HCC cells, where the knockdown of each of the three AKT isoforms reduced lactate production.

Moreover, we also found that the knockdown of AKT1, AKT2 or AKT3 can decrease the OCR, and the activation of each of the three AKT isoforms can increase OCR in HCC cell lines. Consistently, it has been reported that the inhibition of AKT signaling can decrease the OCR in gastric cancer and esophageal cancer [26,27]. Vice versa, the activation of AKT has been shown to increase the OCR in different types of cancer cell lines such as Rat1a, HEK293, glioblastoma cell line U251 and ovarian cancer cells lines TOV112D and TOV21G [28,29], in line with our results in HCC presented in this study.

Current data suggest an association between upregulated mitochondrial metabolism and carcinogenesis, but the potential molecular mechanisms are still unclear. PDH is a gatekeeper multiprotein complex that catalyzes the conversion from pyruvate to acetyl coenzyme A (acetyl CoA), thereby regulating mitochondria respiration. Additionally, the dephosphorylation of PDH activates its function, but the phosphorylation of PDH at distinct sites blocks its activity [30,31].As the gatekeeper complex for mitochondria respiration, inhibiting PDH activity by the upregulation of its phosphorylation through the downregulation of PDH phosphatase expression was validated and shown to decrease the OCR in glioblastoma [32]. Our study demonstrates that the knockdown of AKT1, AKT2 and AKT3 can increase the inhibitory phosphorylation of PDH at Ser232, while the expression of activated variants of each of the three AKT isoforms can decrease PDH phosphorylation. These data strongly suggest that the AKT-dependent regulation of the oxygen metabolism occurs via AKT-mediated PDH phosphorylation in HCC cells. Because this phosphorylation of PDH occurs in opposition to the AKT kinase activity (knockdown vs. overexpression of activated mutants), we postulate that AKT phosphorylates another protein which then induces the dephosphorylation of PDH at Ser232. A candidate protein might be the PDH phosphatase, which dephosphorylates PDH at this specific residue.

Moreover, phosphorylation at other residues may also add to the post-translational PDH regulation. Cerniglia et al. reported that the genetic knockdown of AKT1 reduced the oxygen consumption rate in human head and neck cancer cell lines in vitro by 30% to 40% and increased the phosphorylation level of the PDH complex at Ser293, which again suppresses PDH activity [33]. In contrast, phosphorylation at Ser264 was unchanged following the inhibition of AKT1 in the prostate cancer cell line PC-3 [24]. Taken together, while our study strongly suggests that all AKT isoforms negatively affect the activity of PDH through phosphorylation at serine 232 in HCC cell lines, PDH is also regulated by phosphorylation at alternative sites by both AKT and other kinases which might contribute to the metabolic reprogramming towards glycolysis in cancer.

**Figure 9:**
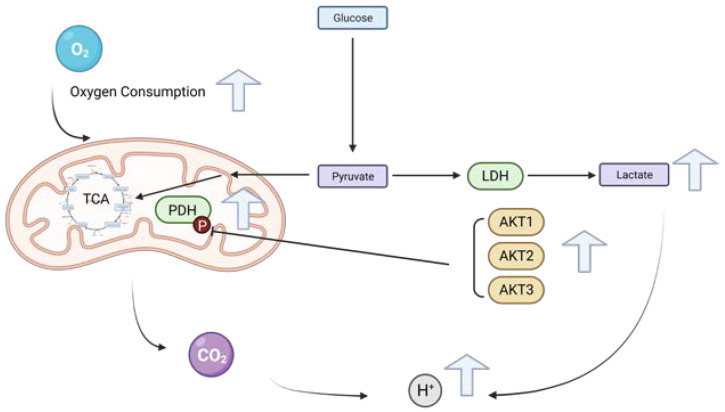
Model of the regulation of oxygen consumption and lactate production by the three AKT isoforms. AKT1, AKT2 and AKT3 increase oxygen consumption in HCC cells by a molecular mechanism leading to dephosphorylation of PDH at the inhibitory regulatory Ser232. The upregulation (blue arrows) of metabolic changes compared with control cells are shown.

In the 1920s, Otto Warburg demonstrated that cultured tumor tissues show high lactate secretion rates, even in the presence of oxygen (aerobic glycolysis), which is also referred to as the Warburg effect [34]. The Warburg effect was observed in tumor cells and other proliferating cells. This augmentation of aerobic glycolysis in cancer cells is frequently thought to occur together with damage to mitochondrial respiration [35]. These classical notions considered that respiration is impaired in highly glycolytic tumor cells. However, our data demonstrate that the knockdowns of each of the three AKT isoforms can also reduce lactate production in Hep3B and Huh7 cell lines (Figure 9). Recently, several studies have suggested that oxidative respiration is not reduced or even increased in a large number of tumor cells despite high levels of aerobic glycolysis [36,37]. Accordingly, there is a widespread agreement with Warburg’s observance that tumor cells have promoted glycolysis; nevertheless, they do still retain oxidative phosphorylation [38]. Moreover, an investigation into hematopoietic and glioblastoma cell lines revealed that the constitutive activation of AKT enables an increased glycolytic rate without changing oxygen consumption [39]. However, as we only analyzed the OCR, ECAR and lactate in HCC cell lines, the other metabolic substrates in the HCC should be confirmed in the future.

Since 2020, for advanced stage HCC, the combination of VEGF inhibitors+ ICI or ICI combination (anti-CTLA + anti-PD-L1) therapy have become the first line therapy. Nonetheless, the usage of novel ICIs and TKIs improve HCC outcomes but the response rates are still low [40].

Drug resistance, which particularly causes the low response rate to HCC treatment, may be attributed to the following reasons. Some TKIs can target several kinase pathways. Consequently, they can also simultaneously or consecutively activate additional regulators and compensatory signaling transduction pathways—for instance, PI3K/AKT and tumor hypoxia—leading to acquired resistance [41]. As for ICIs, they are effective only in a subset of patients due to the many mechanisms that tumors adopt to blunt anti-tumor immunity inflicted by the tumor microenvironment (TME), such as insufficient vascularization, hypoxia, nutrient shortage, lactate accumulation and microenvironment acidification [42]. Sustained sorafenib treatment leads to the reduction in microvessel density, which can promote intratumoral hypoxia [41]. The inhibition of AKT can reduce hypoxia and may thereby reduce the drug resistance of HCC cells. Lactic acid in the tumor microenvironment results in immune cell de-differentiation and the suppression of immune effector cell proliferation [43]. Importantly, the suppression of all three AKT isoforms could reduce acidification in the HCC microenvironment and thereby may decrease the drug resistance against ICIs like atezolizumab. Thus, targeting the distinctive cross-talk between tumor cells and the tumor microenvironment bears as a promising method to restrain HCC progression and to diminish the immunosuppressive pressure mediated by the hypoxic/acidic metabolism, specifically regarding the potential combination of this strategy with the first-line combination of ICI and TKI anti-cancer therapy [44]. The pan-AKT inhibitor capivasertib, an orally bioavailable small-molecule inhibitor of all three AKT isoforms, has been newly approved by the Food and Drug Administration (FDA) for hormone receptor (HR)-positive, HER2-negative locally advanced or metastatic breast cancer, and might also be considered as a future therapeutic approach for HCC treatment [45].

## 4. Materials and Methods

### 4.1. Cell Culture and Culture Conditions

The tumor cell lines Hep3B and Huh7 (human hepatocellular carcinoma) were kindly provided by Prof. Dr. Hans Will from the Leibniz Institute of Virology, Hamburg, Germany. Cell lines were cultured in Dulbecco’s Modified Eagle Medium (DMEM) (#41965-039, Thermo Fisher Scientific Inc., Waltham, MA, USA) supplemented with 10% fetal calf serum (FCS) (#26140-079, Thermo Fisher Scientific Inc., Waltham, MA, USA) and 1% penicillin/streptomycin mix (#15140-122, Thermo Fisher Scientific Inc., Waltham, MA, USA). Cells were maintained at 37 °C in a humidified atmosphere containing 5% CO_2_. All cells were tested for mycoplasma contamination regularly. The cells were passaged twice a week, utilizing trypsin/EDTA solution, washed and resuspended in the respective cell culture medium.

### 4.2. Stable AKT Isoform-Specific Knockdown and Ectopic Expression of Activated AKT Isoforms

pLKO.1-puro vectors encoding either AKT1, AKT2, AKT3 or scrambled shRNA were purchased from Sigma-Aldrich (Taufkirchen, Germany). Activated pseudophosphorylated mutations (AKT-DD) of each AKT isoform (e.g., AKT1-T308D/S473D) were constructed by site-directed mutagenesis and stably expressed in Hep3B and Huh7 cells by lentiviral transduction. The generation of pseudotyped lentiviral particles and cell transductions were performed as previously described [15]. Transduced cells were selected with puromycin (Sigma-Aldrich, Taufkirchen, Germany) in DMEM medium (final concentration: 1.5 μg/mL).

### 4.3. Western Blot Analysis and Densitometric Quantification

The tumor cell lysates were prepared in NP40 lysis buffer containing Tris 1% NP-40 (#98379, Sigma-Aldrich, St. Louis, MO, USA), 2% Aprotinin (#A162.2, Carl Roth GmbH + Co. KG, Karlsruhe, Germany), 2 mM EDTA (#E5134, Sigma-Aldrich, St. Louis, MO, USA), 50 mM NaF (#S7920, Sigma-Aldrich, St. Louis, MO, USA), 10 mM NaPPi (#S6422, Sigma-Aldrich, St. Louis, MO, USA), 10% Glycine (#3908, Carl Roth GmbH + Co. KG, Karlsruhe, Germany), 1 mM Sodium orthovanadate (#S6508, Sigma-Aldrich, St. Louis, MO, USA) and 1 mM PMSF (#10837091001, Sigma-Aldrich, St. Louis, MO, USA). Thereafter, the proteins were blotted onto a nitrocellulose membrane and incubated with specific primary antibodies against AKT1 (#2938S, Cell Signaling Technology Inc., Danvers, MA, USA), AKT2 (#5239S, Cell Signaling Technology Inc., Danvers, MA, USA), AKT3 (#8018S, Cell Signaling Technology Inc., Danvers, MA, USA), PDH (#2784S, Cell Signaling Technology Inc., Danvers, MA, USA), p-PDH Ser232 (#15289S, Cell Signaling Technology Inc., Danvers, MA, USA) and GAPDH (#G0622, Santa Cruz Biotechnology Inc., Dallas, TX, USA). Afterwards, the membrane was incubated with the appropriate secondary antibodies against mouse-IgG (#7076, Cell Signaling Technology) or rabbit-IgG (#7074, Cell Signaling Technology). The protein expression was analyzed using the LAS-4000 Imager from Fuji (Raytest, Straubenhardt, Germany). Densitometric quantification was carried out using AIDA Image Analyser Software Version 3 (Elysia-raytest GmbH, Straubenhardt, Germany).

### 4.4. Seahorse Metabolic Flux Measurement

The XF-96 Extracellular Flux Analyzer (Agilent Technologies, Santa Clara, CA, USA) was used to determine the OCR and ECAR. Briefly, 20,000 Hep3B and Huh7 cells per well were plated in XF96-well microplates (Agilent Technologies, Santa Clara, CA, USA) with Dulbecco’s Modified Eagle Medium (DMEM) (#41965-039, Thermo Fisher Scientific Inc., Waltham, MA, USA) supplied with 10% fetal calf serum (FCS) (#26140-079, Thermo Fisher Scientific Inc., Waltham, MA, USA) and 1% penicillin/streptomycin mix (#15140-122, Thermo Fisher Scientific Inc., Waltham, MA, USA) as well as 1.5 µg/mL Puromycin. Then, the cells were allowed to settle down at 37 °C, 5% CO_2_ overnight. The hydrate cartridge (Agilent Technologies, Santa Clara, CA, USA) added 200 µL ddH_2_O per well and then the cartridge was maintained at 37 °C in a CO_2_-free incubator overnight. Three hours before the OCR and ECAR determinations, the hydrate cartridge added 200 µL XF Calibrant (#100840 Agilent Technologies, Santa Clara, CA, USA) per well and was then maintained at 37 °C in a CO_2_-free incubator. One hour before OCR and ECAR determination, the cell culture medium was carefully removed, wells were washed with 200 µL of assay medium, and, finally, 200 µL of assay medium was added. The assay medium consisted of XF DMEM medium pH 7.4, 500mL (#103575-100 Agilent Technologies, Santa Clara, CA, USA) supplemented with 1 mM sodium pyruvate (#S8636, Sigma Aldrich, St. Louis, MO, USA), 2 mM glutamine (#25030-081, Thermo Fisher Scientific Inc., Waltham, MA, USA), and 10 mM glucose (#A24940-10, Thermo Fisher Scientific Inc., Waltham, MA, USA). Cells in XF96-well microplates were cultured for 60 min at 37 °C in a CO_2_-free incubator before the start of the measurement. During OCR and ECAR analysis, cells were treated with 1.0 µM oligomycin (#75351, Sigma Aldrich, St. Louis, MO, USA), 0.5 µM fluorocarbonyl cyanide phenylhydrazone (FCCP) (#15218, Cayman Chemical, Ann Arbor, Michigan, USA), 0.5 µM rotenone (#13955, Cayman Chemical, Ann Arbor, Michigan, USA) and 0.5 µM antimycin A (#A8674, Sigma Aldrich, St. Louis, MO, USA). OCR and ECAR data were calculated using the software (Wave Desktop and Controller 2.6, Agilent Technologies, Santa Clara, CA, USA) provided by the manufacturer.

### 4.5. Bradford Assay for Normalization of Seahorse Assay Data

The normalization of seahorse assay was conducted by Bradford assay. To prepare the cell samples for the seahorse measurement, all media from the wells of the seahorse plate were removed by aspiration. The wells of the seahorse plate were gently washed with 100 µL PBS, and thereafter, the PBS was completely aspirated. Then, 20 µL of lysis buffer containing 0.1% Triton X-100 (#T8787, Sigma Aldrich, St. Louis, MO, USA) was added to each well. Empty seahorse wells served as controls to evaluate a potential interference of the lysis buffer in the protein assay. Bradford Reagent (#500-0006, BioRad, Hercules, CA, USA) was diluted in a ratio of 3:10 in ddH_2_O. Then, 180 µL of diluted Bradford reagent was added to each well of the seahorse plate and mixed well. The total volume in each well was 200 µL. The absorbance was measured at 575 nm by using a microplate reader (Tecan, Männedorf, Switzerland). Thereafter, the zero-protein blank average was subtracted from the absorbance of all wells. The protein concentration of each well in the seahorse assay was calculated based on the absorbance value from the microplate reader.

### 4.6. Lactate Determination

Hep3B and Huh7 cells were plated at a density of 5000 cells/well into a 96-well flat-bottomed cell culture plate (Greiner Bio-One, Kremsmünster, Austria). For Hep3B and Huh7 cells’ cultivation in this assay, Dulbecco’s Modified Eagle Medium (DMEM) (#41965-039, Thermo Fisher Scientific Inc., Waltham, MA, USA) supplied with 10% dialyzed fetal calf serum (d-FCS) (#A3382001 Thermo Fisher Scientific Inc., Waltham, MA, USA), 5 mM glucose (#A24940-10, Thermo Fisher Scientific Inc., Waltham, MA, USA), 2 mM glutamine (#25030-081, Thermo Fisher Scientific Inc., Waltham, MA, USA) and 1% penicillin/streptomycin mix (#15140-122, Thermo Fisher Scientific Inc., Waltham, MA, USA) was used. At the indicated time points, 2.5 µL of the medium from the well was collected, diluted in 97.5 µL PBS and stored at −20 °C. According to the technical manual from Lactate-Glo Assay (J5021, Promega, Madison, WI, USA), 25 µL of the sample and 25 µL lactate detection reagent was mixed in 384-well plates (Greiner Bio-One, Kremsmünster, Austria) and incubated for 60 min at room temperature. The luminescence was measured using a microplate reader (Tecan, Männedorf, Switzerland).

### 4.7. Statistics

All statistical analyses were performed with implemented functions in GraphPad Prism 8.4.1 (GraphPad Software Inc., San Diego, CA, USA). Statistical significance was tested using unpaired two-tailed Student’s t-test when comparing two groups and using one-way ANOVA with Dunnett’s post hoc test when comparing more than two groups. Results were considered significant if *p* < 0.05. *p* values were encoded into asterisks, as follows: * *p* ≤ 0.05; ** *p* ≤ 0.01; *** *p* ≤ 0.001.

## 5. Conclusions

In summary, our data demonstrate that the targeting of a single AKT isoform was sufficient to inhibit the oxidative respiration and lactate production in HCC cell lines, underlining the essential role of all three isoforms in the metabolic regulation in HCC tumor cells. By reducing oxygen consumption and simultaneously reducing lactate production, AKT inhibition could help to reduce tumor hypoxia and the acidification of the tumor microenvironment. Therefore, AKT could be a valuable target to reduce or even reverse pathological changes in the metabolism of HCC patients, which may be useful in combination with other therapeutic approaches.

## Data Availability

Data contained within the article.

## Supplementary Materials

The supporting information can be downloaded at: https://www.mdpi.com/article/10.3390/ijms25042168/s1.
