# Supplementary material for: All Three AKT Isoforms Can Upregulate Oxygen Metabolism and Lactate Production in Human Hepatocellular Carcinoma Cell Lines
Source: Int J Mol Sci. 2024 Feb 11;25(4):2168. doi: 10.3390/ijms25042168 (PMC10889766; doi:10.3390/ijms25042168)
Supplement: Supplementary file 1 [file ijms-25-02168-s001.zip › ijms-2849967-supplementary.pdf]

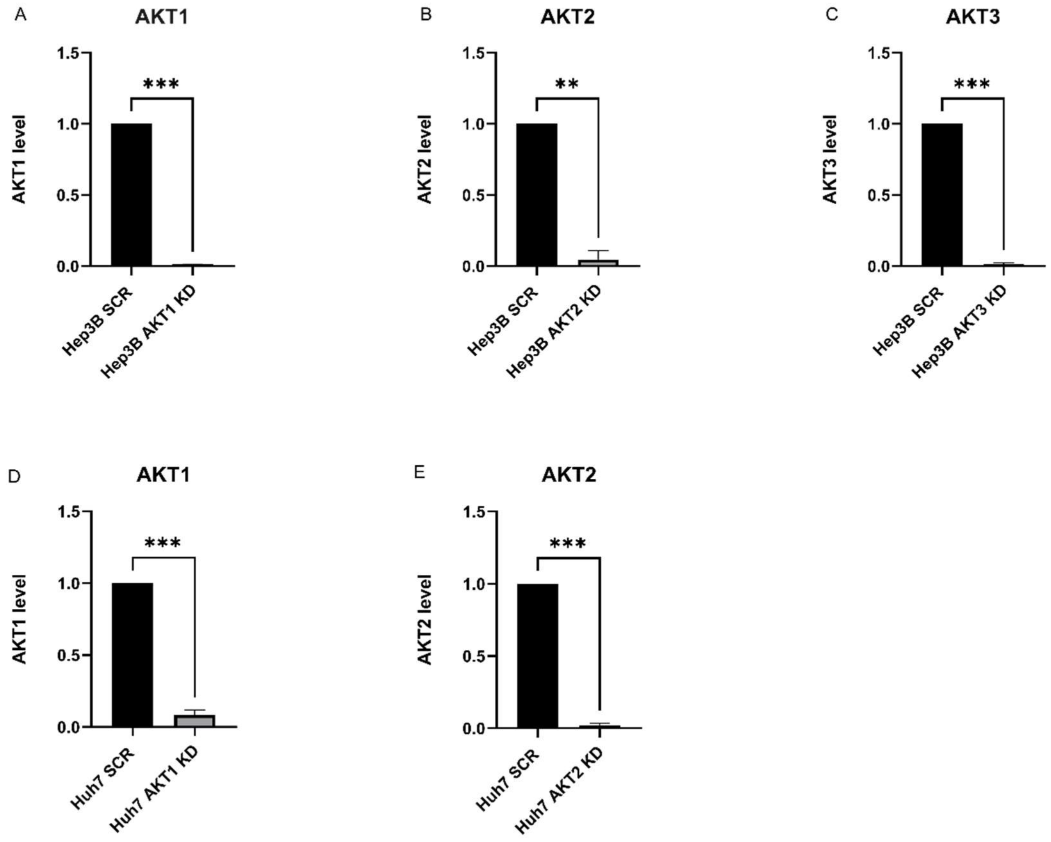

Supplementary Figure S1 Relative levels of knockdown of AKT1, AKT2 and AKT3 in HCC cell lines. Lentiviral mediated knockdowns of AKT isoforms in Hep3B and Huh7 cell lines were compared to the parental cells by Western blot analysis. **(A-C)** Quantification of AKT isoform performed from Western blot analysis of triplicates and normalized to intensity of Hep3B parental cells signals. **(D-E)** Quantification of AKT isoforms performed from Western blot analysis of triplicates and normalized to intensity of Huh7 parental cells signals. Bars indicate mean with SD; ** = $p < 0.01$; *** = $p < 0.001$..

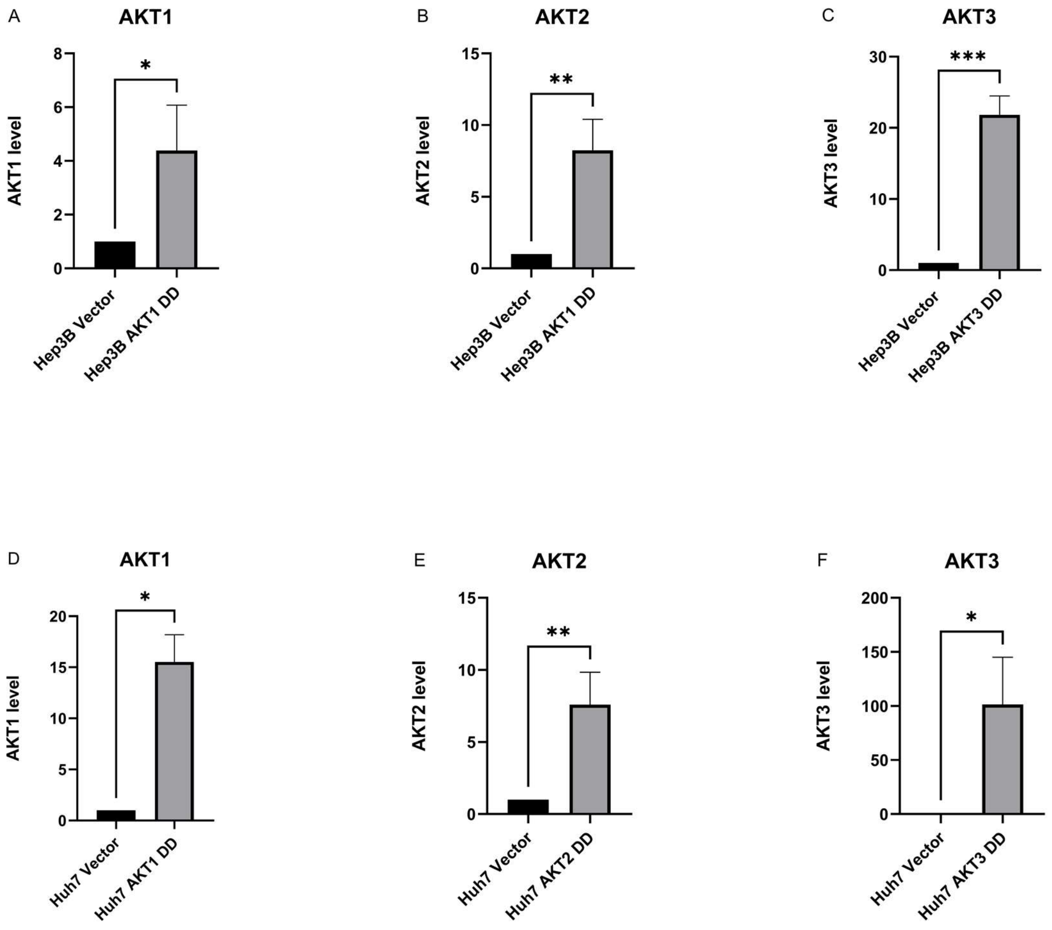

Supplementary Figure S2 Relative levels of overexpression of AKT1, AKT2 and AKT3 in HCC cell lines. Hep3B and Huh7 cells were transduced with lentiviral vectors with the constitutively activated AKT isoforms (AKT-DD) and overexpression was compared to HCC parental cells. Expression levels were quantified by Western blot analysis of triplicates. **(A-C)** Quantification of AKT isoform overexpression in Hep3B cells **(D-F)** Quantification of AKT isoform overexpression in Huh7 cells. Bars indicate mean with SD; * = $p < 0.05$; ** = $p < 0.01$; *** = $p < 0.001$..

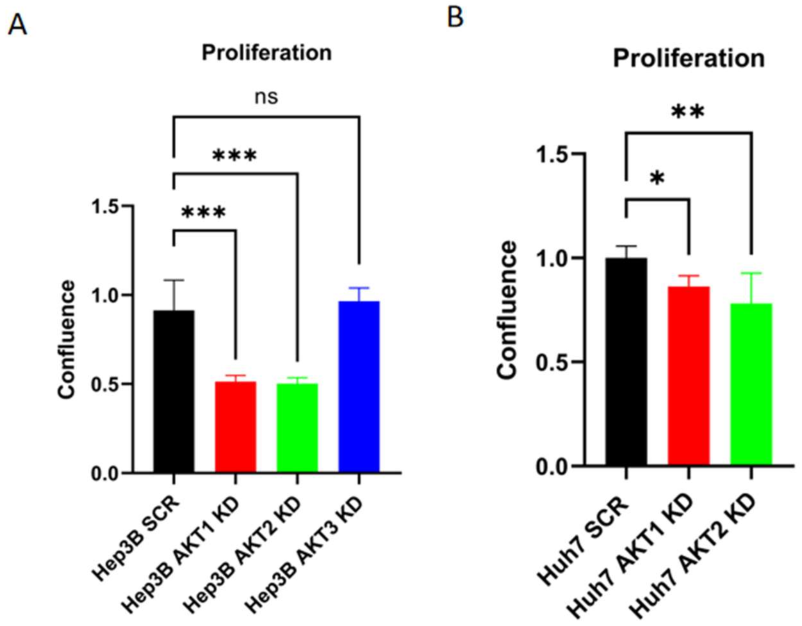

Supplementary Figure S3 Effects of knockdowns of AKT isoforms on the proliferation of HCC cells. (A) Hep3B cells harboring AKT isoform knockdowns were seeded in a 96-well plate and confluence was monitored with the IncuCyte live cell imaging system. (B) Huh7 cells with AKT isoform knockdowns were seeded in a 96-well plate and confluence was monitored with the IncuCyte live cell imaging system. Bars indicate mean with SD; * = $p < 0.05$, ** = $p < 0.01$.*** = $p < 0.001$
